# MRPS16 Regulates NFATC2 Through the Wnt/β‐Catenin Pathway to Promote Glioma Proliferation

**DOI:** 10.1111/jcmm.71027

**Published:** 2026-01-23

**Authors:** Xudong Li, Shaojie Yu, Minjie Wang, Zihan Gong, Qihong Cheng, Xuan Wang, Xiaobing Jiang

**Affiliations:** ^1^ Department of Neurosurgery, Union Hospital, Tongji Medical College Huazhong University of Science and Technology Wuhan Hubei China

**Keywords:** β‐Catenin, glioma, *MRPS16*, *NFATC2*, Wnt

## Abstract

There have been reports that overexpression of mitochondrial ribosomal protein S16 (MRPS16) can greatly improve the growth of tumour cells, migration and invasion abilities in many ways. However, the role of MRPS16 in glioma cell proliferation, which is closely associated with tumour malignancy, remains unclear. The study applied a human gene expression array to investigate the expression levels of genes within glioma tissues in comparison with normal brain tissue. By RT‐PCR, cell counting, flow cytometry, MTT assays, colony formation and injection of mice, we deeply explored the role of MRPS16 in glioma cell growth and the underlying mechanism. MRPS16 expression was significantly higher in glioma tissues compared with normal brain tissues. In the cultured glioma cells, glioma cell proliferation was inhibited, and cell cycle arrest and cell apoptosis were induced after MRPS16 knockdown. In BALB/c mice inoculated with glioma cells knocked down for MRPS16, it was found that tumour proliferation and growth were relatively slower than the control. Through further prediction and gene transformation of cultured cells, it is confirmed that the presence of MRPS16 promotes the proliferation of glioma cells through the Wnt/β‐catenin/NFATC2 pathway. MRPS16 and NFATC2 promote glioma cell proliferation, which was confirmed by in vivo BALB/c mice inoculation. The Wnt/β—Catenin/NFATC2 pathway plays a role in promoting glioma cell proliferation by MRPS16, which is shown in our experimental data. Inhibition of MRPS16 may be a promising and effective treatment option for gliomas.

## 1 Introduction

Glioma is the most common and lethal primary tumour in the brain, accounting for 50%–60% of all neurological malignancies [1]. The etiologic features of this tumour are still not well known. Due to resistance to radiotherapy and chemotherapy, the average survival time of patients is less than 12 months [2]. In recent years, due to the combined application of radiotherapy, chemotherapy and targeted therapy for specific gene loci, the long‐term survival rate of patients after surgery has been significantly improved [3]. However, the outcome is still not ideal. Glioma cell proliferation is correlated with a high degree of tumour malignancy, but the main mechanism of its proliferation is still unknown [4]. Therefore, in order to find new high‐efficiency therapies for glioma, it is necessary to understand the specific mechanisms through which glioma cells proliferate and grow, and find new therapeutic targets.

Mitochondrial ribosomal protein S16 (MRPS16) is the small mitochondrial ribosomal protein at the core of the mitochondrial respiratory chain [5]. MRPS16 is a 137–amino acid mitochondrial ribosomal protein that plays an essential role in mitochondrial protein synthesis and cellular energy metabolism. MRPS16 is a very well conserved ribosomal protein from yeast to mammals' mitochondria. Human mitochondrial ribosomes consist of two different subunits, the smaller ribosomal subunit and the larger ribosomal subunit, each of which contains some type of rRNA and protein [6, 7] mitochondrial ribosomal proteins' mutations will trigger serious respiratory chain dysfunction. MRPS16 has nonsense mutants in homozygote form due to corpus callosum and brain abnormalities, fatal neonatal lactic acidosis [8, 9]. MRPS16 was reported to promote tumour progression by the PI3K/AKT/Snail pathway in glioma [10]. Regarding the effect of MRPS16 on glioma, no other research has mentioned this. The role of MRPS16 in glioma development has not yet been fully elucidated.

Wnt proteins are lipid‐modified and secreted glycoproteins and act as a growth factor [11], and allow for communication between cells. The Wnt signalling pathway includes Notch‐Delta, Hedgehog, transforming growth factor β (TGF‐β)/bone morphogenetic protein (BMP), Hippo and β‐Catenin [12] to regulate the developmental processes [13]. Wntproteins can raise β—Catenin levels and enhance signal transmission within the cells. The inhibition of phosphor‐β‐Catenintublin causes both saturated‐phosphorylated‐β‐Catenintublin and new β‐Catenintublin to accumulate, which is then transferred into the nucleus for the activating of target gene [14, 15]. The nuclear factor that activates T cells (NFAT) transcription family regulates the core genes in many developmental systems by combining chromatin with other transcription factors and co‐activators to further integrate multiple signalling pathways [16]. In many cancers, such as breast cancer and melanoma, NFAT has been shown to be involved in cell proliferation, invasion, migration, angiogenesis, etc. [17, 18]. NFATC2 (NFAT, CYTOPLASMIC 2) belongs to the NFAT family and is mainly distributed in the cytosol. NFATC2 is able to transmit inside nucleus in response to T cell receptor's stimulation. Also it may become members of the nucleus transcription complex. Gene transcription and T cells getting turned on can also be brought about by this as well. Or, it may just mean that there isn't any NFATC2. There is way too many lymphocytes growing and it is hard for them to make cartilage [19, 20]. It tumour progression and cell apoptosis were also found both to be linked strongly with the Wnt/β‐cat—ten/NFAT signalling [21, 22]. If there is any effect on the proliferation of glioma cells from this path need to be investigated.

In the current study, it was found that MRPS16 shows notably high expression within human glioma tissue samples. Knockdown of MRPS16 can slow down the growing speed of glioma cells (such as U87 or U251 Cells), help them die. Glioma cells proliferating induced by MRPS16 may involve Wnt/β − Catenin/NFATC2 signaling pathway. Therefore, MRPS16 might be an aim for addressing gliomas.

## 2 Materials and Methods

### 2.1 Patients

With the approval of the ethics committee of the Wuhan Union Hospital (number 2020IEC‐J(482)), the experiment was carried out in accordance with the relevant ethical regulations. Before these patients participated in our study, we asked for the permission of the written form of informed consent from them or their families. In Union Hospital, six surgically excised tumour samples and three autopsy control brain tissues were obtained from January to June 2020. The patients were treated with radiotherapy or chemotherapy after surgery. And finally, all samples were promptly sealed in −80°C liquid nitrogen. Glioma was diagnosed by H&E staining.

### 2.2 Chip Analysis

Total RNA was extracted from the tissues using TRIzol Reagent (Sigma) as per the manufacturer's instructions. RNA concentration and quality were determined using the NanoDrop 2000 (Thermo Fisher Scientific) and Agilent Bioanalyzer 2100 (Agilent). The GeneChip 3′IVT Express Kit (ThermoFisher) and Affymetrix GeneChip PrimeViewTM Human Gene Expression Array (ThermoFisher) were used for microarray expression analysis. Briefly, after first‐ and second‐strand cDNA synthesis, labelled cRNA synthesis by in vitro transcription, cRNA purification and cRNA fragmentation, cartridge array hybridization on the GeneChip Instrument was performed. We used the GeneChip Fluidics Station 450 from ThermoFisher to carry out the automatic washing and staining processes. The cartridge array was scanned with a GeneChip Scanner 3000 (Affymetrix). The predictive impact of MRPS16 on glioma proliferation has been investigated with the employment of the cancer genome atlas (TCGA) database.

### 2.3 Cell Lines and Cell Culture

The human glioma cells U87, U251 were provided by Shanghai GeneChem Biotechnology Co. Ltd. Incubated at 37°C with 5% CO_2_ and Gibco Dulbecco's modified eagle medium with 10% fetal bovine serum (Ausbian) and supplemented with 100 U/ML penicillin and 100 μg/ML streptomycin in a humidifying incubator. Cells were cultivated in 60 mm dishes till reaching 70%–80% confluence; medium change was done every fourth day to ensure best possible growth conditions.

### 2.4 Plasmid Construction and Transfection

MRPS16 cDNA (GenBank accession number: Subcloning of NM_016065 and NM_173091; Figure 2, NFATC2; NM_173091) were transfected into the pGCSIL‐GFP vector to yield the over‐expression plasmid vectors, pGCSIL‐GFP‐MRPS16 and pGCSIL‐GFP‐NFATC2, which were made by Shanghai GeneChem CO. LTD, China.

As mentioned in reference [23], transfection was carried out for both U87 and U251 cells. According to manufacturer Corning, Cells were planted in 48‐well plates at density of 1 × 10^4^ cell/well to reach 70% confluence, followed by transfection in virus‐packaged plasmid: Media was replaced 24 h later.

The short hairpin RNA (shRNA) was designed based on the MRPS16 and NFATC2 sequences individually. According to the design principle of shRNA, we designed the MRPS16 gene‐specific oligonucleotide sequence (5′‐CACCTCTCTAAGCCTATGGAA‐3′) and NFATC2 gene‐specific oligonucleotide sequence (5′‐GAGTCCAAAGTTGTGTTTA‐3′) and confirmed no homology sequence of the same species after BLAST analysis. The oligoduplexes were cloned into pGCSIL‐GFP cloning vectors and then transfected into U87 and U251 cells. Silencing of other genes (OIP5, NBPF15, etc.) was the same as that of MRPS16. The oligonucleotide sequences designed are shown in Table 1.

**TABLE 1:** The oligonucleotide sequences of shRNAs.

GENE	Oligonucleotide sequences of shRNAs
MRPS16	5′‐CACCTCTCTAAGCCTATGGAA‐3′
NFATC2	5′‐GAGTCCAAAGTTGTGTTTA‐3′
OIP5	5′‐ATCAGAGATGGATATTCAA‐3′
NBPF15	5′‐GTTCCAGATGGGAGTCATA‐3′

### 2.5 Cell Counting and MTT Assay

The cells in the logarithmic growth stage, after being transfected, were diluted to a concentration of 2 × 10^3^ cells/well and then seeded into the 96‐well plate. Celigo (Nexcelom): Using Celigo, systematically counted GFP expressing cells over a continuous 5 day period. Then its cell growth curve is as follows:

Cells were plated on 96‐well plates. MTT was used at a final concentration of 0.5 mg/mL and added from day 1, day 2, day 3, day 4, to day 5 post‐seeding. Then, incubate the plates at 37° for 4 h, substitute the medium with DMSO. Then the absorbance at 490 nm was determined by using the MultiscanMk3Microplate Reader from Thermo Fisher.

### 2.6 RT‐PCR

Transcribed RNA into cDNA by using the procedure set out by Promega. Table 2 shows the primers used for the mRNAs and GAPDH. The reaction mixture was held at 42°C for 60 min and then 95°C for another 5 min. Total reaction volume at 12 μL, PCR was conducted using 6 μL of 2× SYBR Green PCR Master Mix, including 0.6 μL of cDNA, 0.3 μL of primers, while making it up to 5.1 μL by adding deionised water. Amplification protocol: 10 min at 95°C; 45 cycles of 5 s at 95°C, 30 s at 63°C and 30 s at 72°C; 5 min at 72°C. Afterwards, the software LightCycler480 was used to generate its respective melting curve as well as to perform real‐time data acquisition and analysis in terms of quantity. I normalised by dividing the data with the GAPDH, using a $2^{-∆∆C_{q}}$; all 3 experiments are carried out.

**TABLE 2:** The primers for RT‐PCR.

Gene	Upstream primer sequence	Downstream primer sequence	Amplified fragment size(bp)
GAPDH	TGACTTCAACAGCGACACCCA	CACCCTGTTGCTGTAGCCAAA	121
NFATC2	CACGGTGGATAAGGACAAGAG	GTGCTGAGGCTGACTTCG	139
MRPS16	GGGGCCACTTAACCATCCG	TGGGACACTTGTTGTGAGCAG	87

### 2.7 Western Blot

After harvest of the cells, cell lysates were extracted from harvested cells and protein concentration was determined using BCA Protein Assay Kit (ThermoFisher). Electrophorese an ali of lysate in 12% SDS—ployacrylamide gel, then tranfer the separate to PVDF membran. After the blocking of membranes by 5% nonfat milk, they were incubated with the respective primary antibody (Table 3). Then the membranes were washed three times with TBST (0.1% (v/v)Tween—20, TBS, pH 7.4), and then incubated with the corresponding secondary antibody (Abcam). After TBST washing for three times, membranes was detected enhanced chemiluminescence plus (ECL+) kit (ThermoFisher) for the immuno‐reactive band detection. We exposed the membranes to X‐ray film to visualise the protein bands on the membrane for further quantification with the use of Quantity One 1D analysis software v. 4.5.2 (Bio‐Rad, USA).

**TABLE 3:** The antibody list.

Name	Source	Company	Catalogue No	MW (kDa)
mTor	Rabbit	CST	#2972	289 kDa
p‐akt(t308)	Rabbit	CST	#13038	60 kDa
GSK‐3β	Mouse	ABCAM	ab93926	47 kDa
MRPS16	Rabbit	Sigma	HPA050081	16 kDa
β‐catenin	Rabbit	CST	9562 s	92 kDa
p‐akt(s473)	Rabbit	CST	#4060	60 kDa
NFATC2	Mouse	R&D	MAB6499	135 kDa
GAPDH	Mouse	SANTA CRUZ	sc‐32233	36 kDa

### 2.8 Cell Cycle Analysis

Cells were plated into 60 mm dishes at 5 × 10^5^ cells per well. Incubate for 5 days after that, then harvest all cells above 10^6^ and wash them three times with ice‐cold D‐Hanks (pH = 7.2–7.4). After being centrifuged at 1300 r/min for 5 min, cells were fixed in 75% precooled ethanol (4°C) for at least 1 h. Following the cells' wash step, the solution was changed again and the cells were stained with a D‐Hanks solution containing 50 mg/mL PI (Sigma). DNA content detection was carried out via a flow cytometer (FACS vantage SE) and cell cycle distribution analysis with ModFit LT software (Verity Software House). It was carried out 3 times.

### 2.9 Flow Cytometric Analysis of Cell Apoptosis

Apoptotic cells were detected using Annexin V—APC staining according to the manufacturer's guidelines (ThermoFisher). In 60 mm dishes, we put cells into the well with a number of 5 × 105. After 5 days, they were then treated with the apoptosis inducer (10 μM camptothecin) for 4 h. Following which, the cells, over 5 × 10^5^ in number, were isolated utilising 0.25% trypsin, then rinsed with cold D‐Hanks solution. The cell pellets were resuspended in 1× binding buffer and then added to Annexin V—APC and incubated at room temp in the dark for 10–15 min. The percentage of the number of apoptosised cells was recorded right away when the stained cells had been run by the FACS Experiment in triplicate or more.

### 2.10 Animal Treatment and In Vivo Imaging Detection

Female BALB/c nude mice, 4 weeks old, were provided by Shanghai Lingchang Biological Technology Co. Ltd., and after receipt, these mice were kept under SPF conditions inside the animal care facility of Shanghai GeneChem Co. Ltd. In the context of animal transplantation, luciferin‐labelled U87 or U251 cells at a concentration of 2 × 10^7^/mL were carefully diluted and then injected into the abdomen or brain tissue of mice. Mice were randomly assigned to experimental groups (*n* = 6 per group), and 5 × 10^6^ cells were injected into each mouse. All in vivo experiments, including subcutaneous and intracranial tumour models, were approved by the Animal Welfare and Research Ethics Committee of Tongji Medical College, HUST (Approval No. 2020IEC‐J(482)).

Tumour formation was observed at day 5–20 after transplantation. At 8 weeks after injection, tumours were taken out and arranged on the whiteboard for photography. Body weight and tumour volume and weight were also measured.

In terms of the in vivo imaging, we used the anaesthesia which was injected 0.7% sodium pentobarbital with an intra‐peritoneal dose of 10 μL/g of body weight for the animals. After that, the animals will be placed under the in vivo imaging instrument (Instrument, Company) for more analysis. IVIS Spectrum in Vivo imaging system (Perkin Elmer) was used for analysing the bioluminescence.

### 2.11 Data Analysis

Experiment was done three times using the software SPSS 23.0 and Prism GraphPad 8.0 for statistical analysis; using the student *T* test to determine the statistic in the most tests. Mann–Whitney was used on bioluminescence data. In order to examine the relationship among various variables, spearmans correlation analysis was done as a non‐parametric statistic method. The Kaplan–Meier method was used to make survival curves and the log‐rank test was done to do comparisons. Data are expressed as mean ± s.d. *p* < 0.05 was significant.

## 3 Results

### 3.1 High Expression Level of *MRPS16* in Human Glioma

**FIGURE 1:**
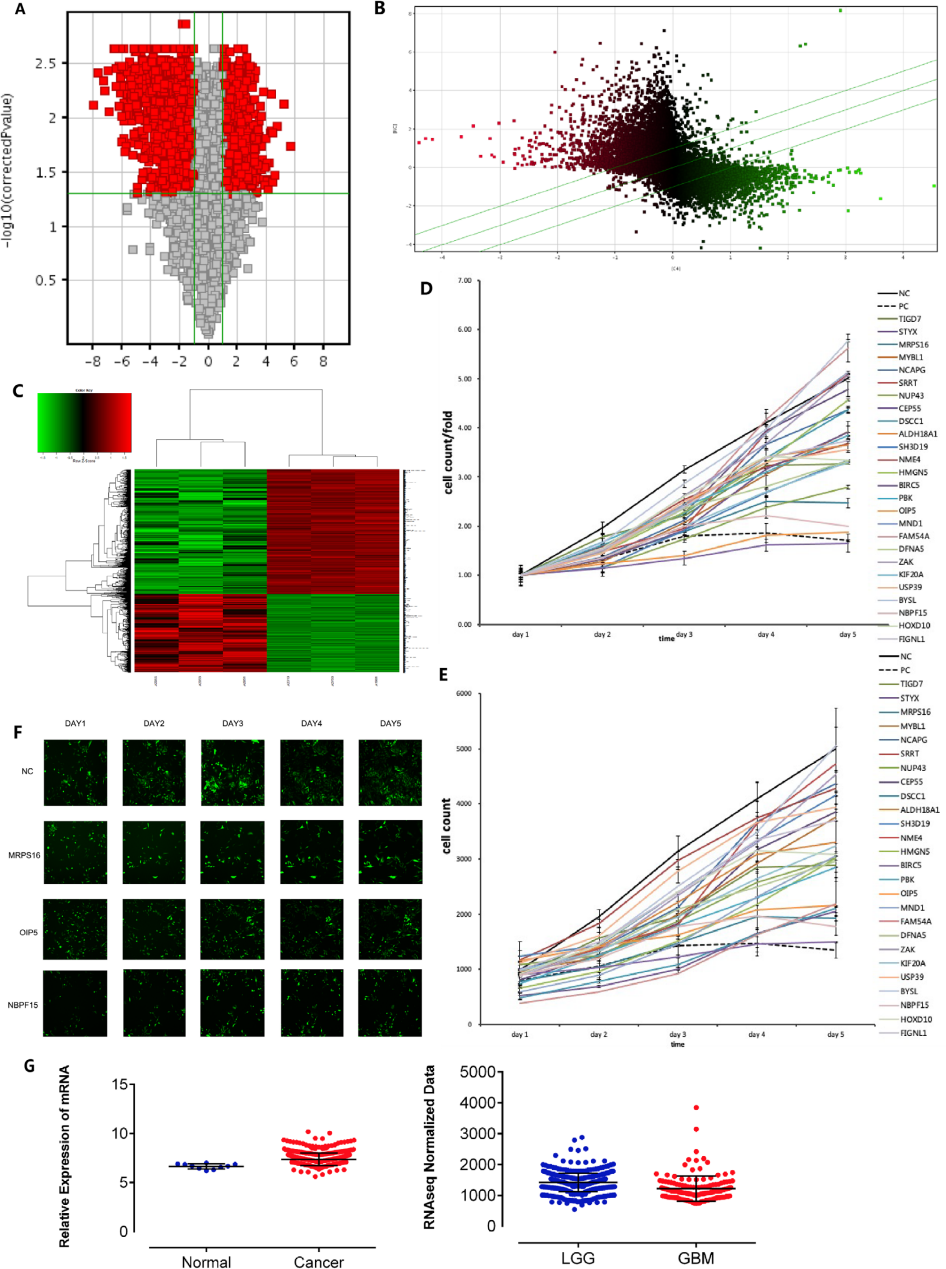
*MRPS16* over‐expression in human glioma. Six cases of grade II and grade IV brain glioma and three cases of normal control tissues were collected. The genes that expressed differentially in glioma and normal control cells were identified by the Affymetrix GeneChip PrimeViewTM human gene expression array. (A) Volcano map of the genes differentially expressed between glioma tissue and normal tissue cells. (B) Scatter plot of the differentially expressed genes. (C) Hierarchical clustering of the differentially expressed genes. (D) U87 cells were counted at different culture times after the knockdown of genes differentially expressed in Gene Chip. (E) Fold change of cell number from panel D. (F) Images of U87 cells at different culture times after silencing of *MRPS16*, *OIP5* or *NBPF15*. (G) Relative *MRPS16* mRNA levels were measured by RT‐PCR in U87 cells under *MRPS16* silencing. (H) *MRPS16* expression levels in normal, cancer and different grade glioma (LGG and GBM) tissues from the TCGA database.

To select some essential proteins in the development and metastasis of gliomas, clinically collected six brain glioma specimens of grade II and four of grade IV with three normal control tissues. Table 4 shows the demographic details of all the participants taking part in the study: H&E staining was used to check the specimens. Using the Affymetrix GenechipPrimeViewTM human gene expression array, genes were successfully identified to be differentially expressed when comparing glioma cells to controls (Figure 1A–C). Compared to the normal group, 3158 genes were shown to be differentially expressed in the glioma tissue, including 1263 that were upregulated and 1895 that were downregulated. To check the effect of the differentially expressed genes on glioma cell proliferation, 26 upregulated genes were chosen to be knocked down in cultured human glioblastoma U87 cells by corresponding shRNA. Cell growth was monitored by cell counting (Figure 1D,E). As the results showed, the knockdown of most chosen genes decreased U87 cell proliferation compared to untreated cells. Among these genes, cells with *MRPS16*, *OIP5* or *NBPF15* knockdown grew much slower than cells knocked down with other genes. Microscopic observation of the cells also showed a lower proliferation rate of U87 cells when the knockdown of *MRPS16*, *OIP5* or *NBPF15* was performed (Figure 1F). *MRPS16* has not been reported to be involved in glioma cell proliferation before. Analysis from the TCGA database also indicated that the *MRPS16* level in cancer is higher than that in control. Statistical significance was assessed using Student's *t*‐test. MRPS16 level is highly elevated in HGG relative to LGG, as can be seen with the level of MRPS16 as shown in Figure 1G. The differential expression of MRPS16 in glioma vs. normal brain tissue seems to be quite obvious. There is much greater expression of MRPS16 in glioma cells:

**TABLE 4:** The demographic characteristics of patients with or without glioma tumour.

No.	Gender	Age	Diagnosis	Classification	Grade
1	Female	58	NC	—	—
2	Female	49	NC	—	—
3	Male	46	NC	—	—
4	Female	43	Pilocytic astrocytoma	IDH mutant	C2
5	Male	53	Glioblastoma	IDH mutant	C2
6	Female	52	Glioblastoma	IDH mutant	C2
7	Male	47	Medulloblastoma	IDH wildtype	C4
8	Female	44	Glioblastoma	IDH mutant	C4
9	Male	45	Astrocytoma	IDH wildtype	C4

Abbreviations: C2, grade II brain glioma; C4, grade IV brain glioma; IDH, Isocitrate Dehydrogenase (NADP(+)) 1; NC, negative control.

### 3.2 *MRPS16* Knockdown Suppressed Glioma Cell Proliferation In Vitro and In Vivo

**FIGURE 2:**
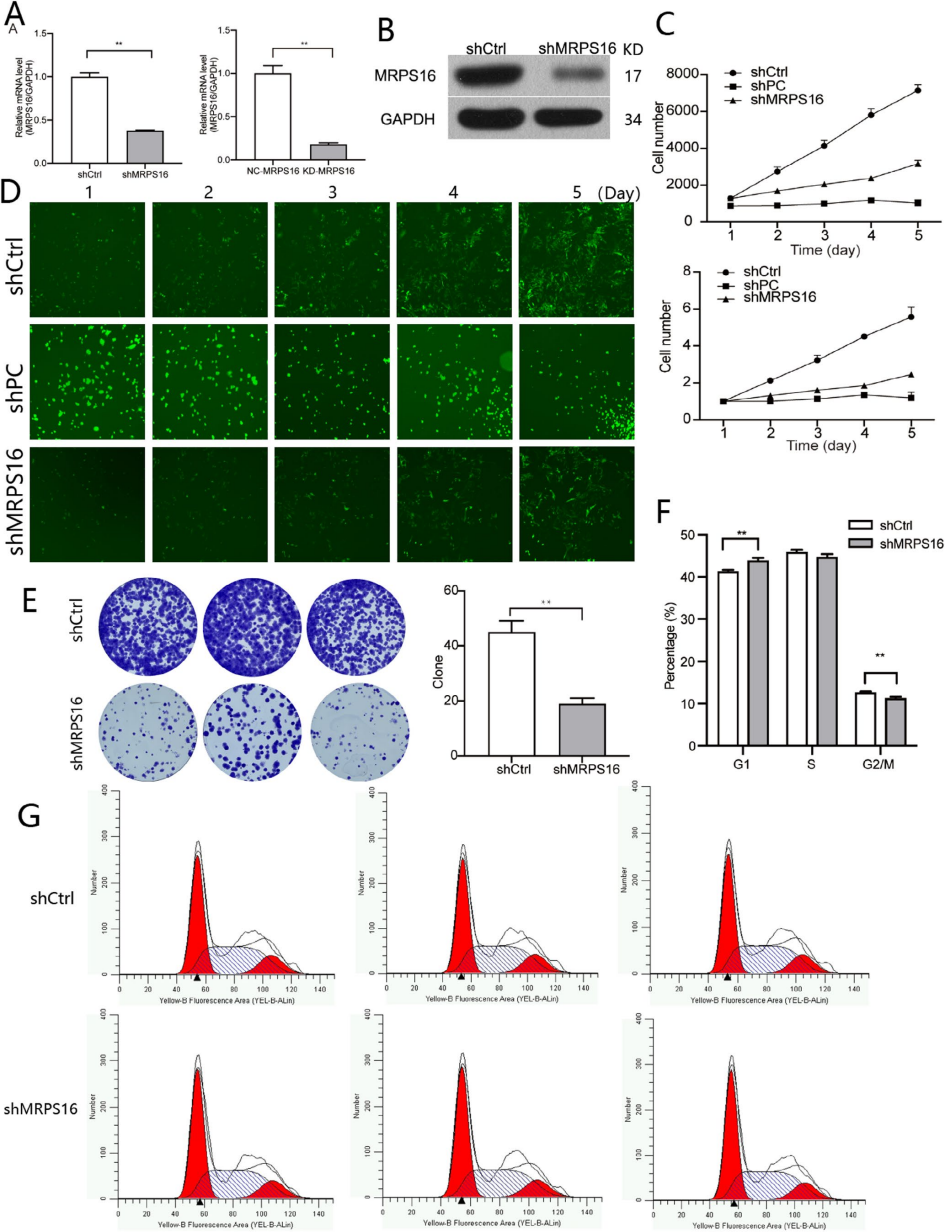
Regulation of *MRPS16* on the proliferation of glioma cells. (A) *MRPS16* was knocked down in U87 cells; *MRPS16* mRNA levels were detected in U87 cells after *MRPS16* knockdown. (B) Western blotting for *MRPS16* protein levels in U87 cells under *MRPS16* knockdown. (C) U87 cell counts at different culture times under *MRPS16* knockdown. (D) U87 cell images at different culture times after knockdown of *MRPS16*. (E) Observation of U87 cell colonies after 23 days under *MRPS16* knockdown. (F, G) U87 cells were collected 5 days after *MRPS16* knockdown and the DNA content was determined by flow cytometry with propidium iodide (PI) staining. The percentage of cells in the G1, S and G2/M phases was measured. **p < 0.01.

In order to study the proliferative effect of MRPS16, we first perform MRPS16 knockdown in U87 cells: The effective inhibition of shRNA on MRPS16 expression can be seen from that the mRNA and protein content of MRPS16 in cells both decreased significantly (Figure 2A,B). The growth of U87 cells was monitored by cell counting. Cell proliferation was significantly decreased when cells were knocked down with MRPS16 gene. It took 5 days for MRPS16‐KD U87 cells to proliferate to the same number as the untreated cells that proliferated for 2 days (Figure 2C). When observed with a fluorescence microscope, the proliferation was also obviously slower in MRPS16‐KD U87 cells than control cells (Figure 2D), Statistical significance was assessed using Student's *t*‐test. Moreover, when looking at the number of clones for 23 days of shRNA knockdown, we can tell that the number of colonies of U87 cells in the group of MRPS16‐KD has been drastically reduced (see Figure 2E), which might mean that MRPS16 could be linked to the ability of U87 cells to produce colonies. The inhibition of proliferation of glioma cells was achieved again through the knockdown of MRPS16.

After silencing MRPS16, which we showed had affected the number of cells, population analysis was performed on U87 Cell Cycle; As seen from Figure 2F and Figure 2G, we noted a much larger quantity of MRPS16‐KD U87 in G1 phase as compared to control cells. On the contrary, the ratio of cells in G2/M phase is quite less (*p* < 0.05), Statistical significance was assessed using Student's *t*‐test. The percentage of MRPS16‐KD U87 cells in the S Phase was similar to that of the Control Cells. These results show that MRPS 16 is related to the cell cycle distribution of glioma U87 cell. If there is more cell in dividing phase, it means tumour cell mitisis is increased. Knockdown of MRPS16 results in fewer cells dividing so it successfully stops cells from growing. It proves that MRPS16 can promote the proliferation of tumour.

**FIGURE 3:**
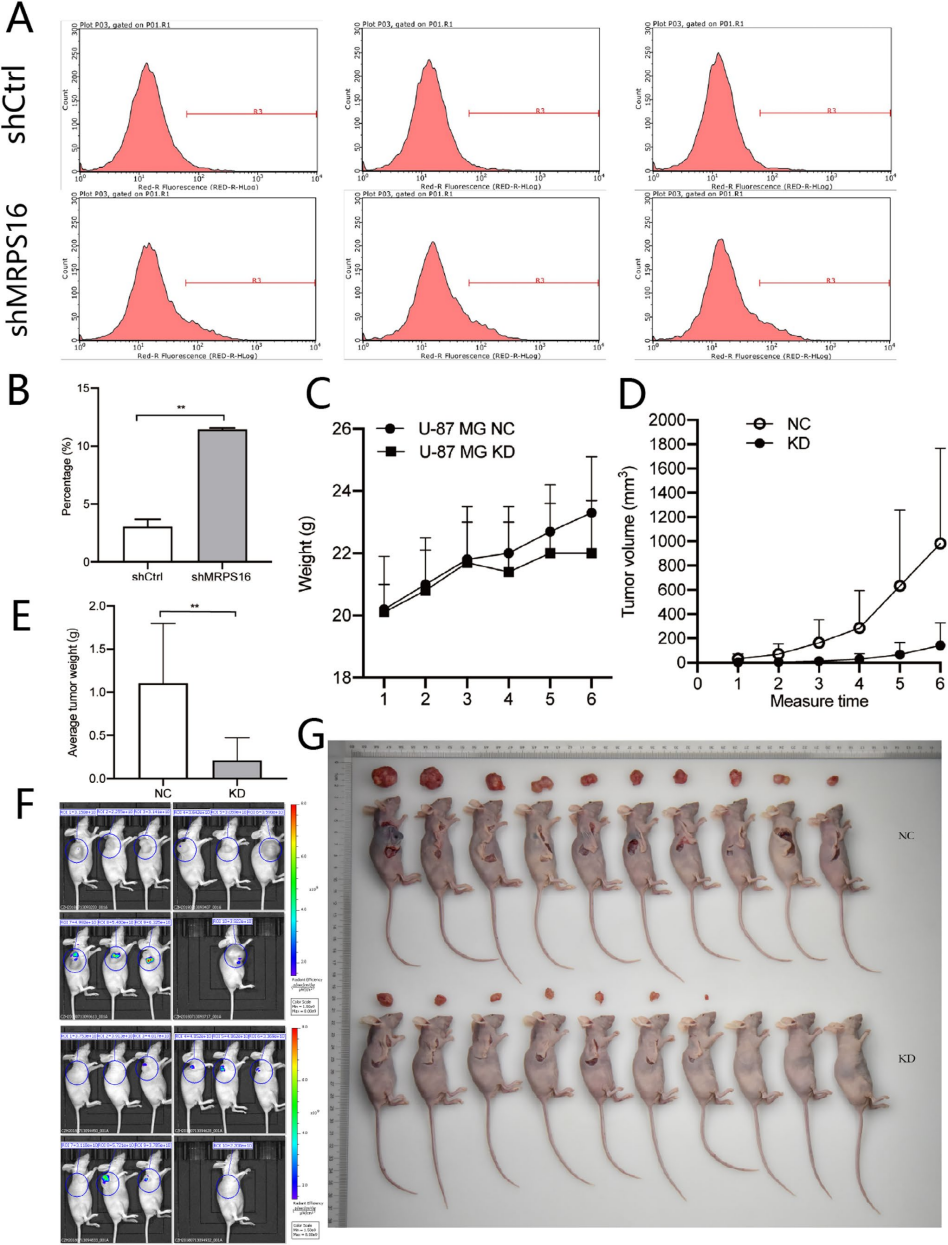
Regulation of *MRPS16* on the apoptosis of glioma cells and animal subcutaneous tumorigenesis experiment. (A, B) U87 cells were collected 5 days after shRNA transduction and subjected to flow cytometry with Annexin V‐APC staining to assess apoptosis. The percentage of apoptotic cells was measured. **p < 0.01. (C) Body weight of BABL/c mice. (D) Tumour volumes in BALB/c mice inoculated with *MRPS16*‐KD U87 cells. (E) Tumour weight in BALB/c mice inoculated with *MRPS16*‐KD U87 cells. (F) In vivo imaging detection of tumours in BALB/c mice. (G) Tumour growth in BALB/c mice inoculated with *MRPS16*‐KD U87 cells.

To determine whether MRPS16 was involved in the cell apoptotic pathway, we used flow cytometry to examine cell apoptosis by Annexin V‐APC staining (Figure 3A,B). After the U87 cells were knocked down following MRPS16 shRNA transduction, the application of the Annexin V‐APC dye resulted in a large increase in the cell population 5 days later, thus demonstrating a significant increase (about 11%) in apoptosis of MRPS16‐KD U87 cells compared to untreated cells (~3%). Statistical significance was assessed using Student's *t*‐test. Knockdown of MRPS16 might make for more comprehensive apoptosis of glioma cells; that is to say, it could increase inhibition by way of regulating cell multiplication.

To assess the effects of MRPS16 knockdown on glioma cell proliferation in vivo, we intraperitoneally injected the MRPS16 knockdown glioma U87 cells into the BALB/c mice and then kept them for a period of 6 weeks. From Figure 2C, comparison between the groups of mice injected with non‐treated glioma cells and the control group shows that there is no considerable difference in body weight. But compared to the cells that were not treated with U87, tumour volume and tumour weight of the mice were much less when the cells infected with MRPS16‐KD U87, Statistical significance was assessed using Student's *t*‐test. As can be seen from Figure 3D,E,G. As was shown via IVIS Spectrum in Vivo imaging system, tumour proliferation presented much less bioluminescence signals in the MRPS16‐KD group as compared with the control group (Figure 3F), Mann–Whitney *U* test was used for bioluminescence data. These results show that MRPS16 knockdown inhibited glioma cell proliferation in vivo.

### 3.3 *MRPS16* Promotes Glioma Cell Proliferation Through *NFATC2*

**FIGURE 4:**
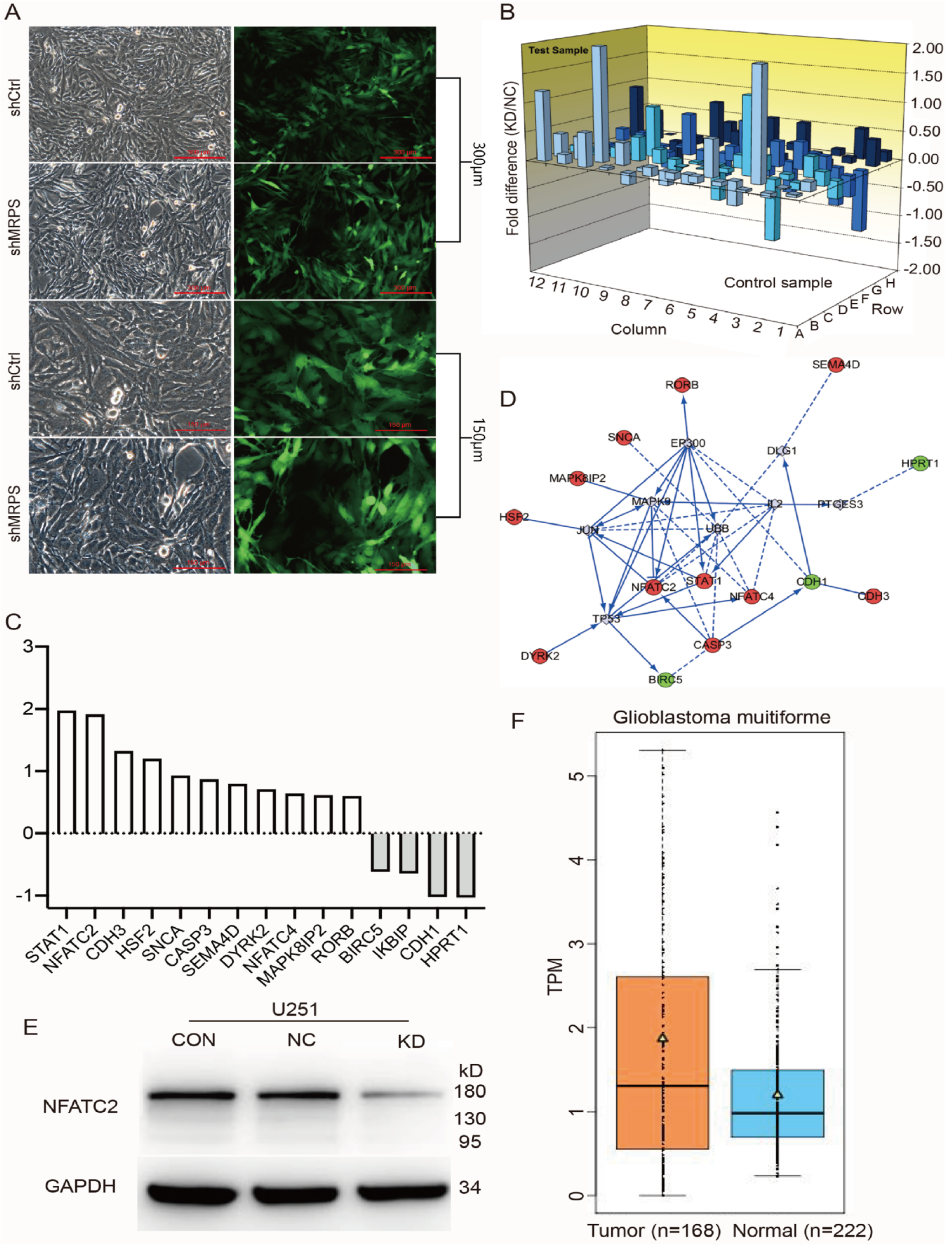
Analysis of *MRPS16* target genes. (A) *MRPS16* was overexpressed in glioblastoma U251 cells. Three days later, U251 cell images were taken. (B) Detection of 96 downstream gene expression levels by RT‐PCR after *MRPS16* knockdown. (C) The expression level of the 15 most differentially expressed genes. (D) Regulation interaction map of the 15 differentially expressed genes. Red indicates up‐regulation and green down‐regulation. Solid arrows indicate definite regulatory relationships and dashed arrows indicate predicted regulatory relationships. (E) Western blotting for *NFATC2* protein in U251 cells under *MRPS16* knockdown. (F) *NFATC2* expression levels in normal and glioma tissues from the TCGA database.

Then we searched for ways MRPS16 helps glioma cells replicate and we wanted to know the exact genes or proteins controlling that. First, by transfection, it is overexpressed in glioma U251 cells. Three days later, cell proliferation was observed to be much faster in *MRPS16*‐transfected U251 cells than control cells (Figure 4A). We next identified the downstream target of *MRPS16*. By RT‐PCR, the expression of 96 downstream genes was measured, some of which are expressed differently (Figure 4B). Fifteen genes related to *MRPS16* were found to show differential expression, among which the expression of *NFATC2* was upregulated much more prominently than other genes (Figure 4C). Through connectivity and regulation analysis of these 15 genes with *MRPS16* and related genes, *NFATC2* was obviously connected and positioned in the center (Figure 4D). The protein level of NFATC2 in MRPS16‐KD U251 cells was lower than that of the control group, as shown in Figure 4E. Furthermore, based on the analysis of the TCGA database, it can be seen from Figure 4F that the expression level of NFATC2 in glioma tissue is significantly higher than that in control tissue. And it is also put forth that NFATC2 takes part in regulating the growth of glioma cells by way of MRPS16, Statistical significance was assessed using Student's *t*‐test.

**FIGURE 5:**
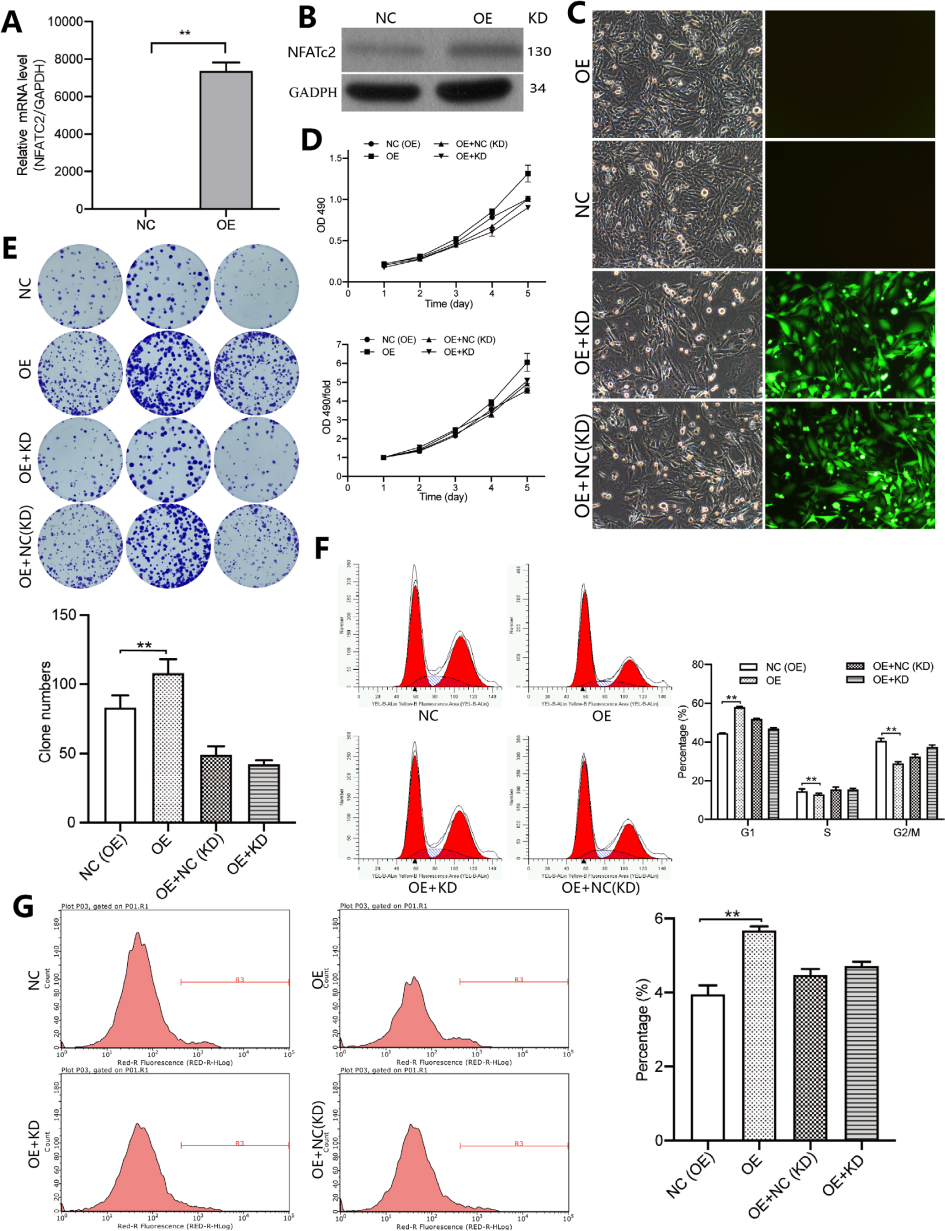
Regulation of *NFATC2* on glioma cell proliferation. NFATC2 was overexpressed in U251 cells using a lentiviral expression vector, and proliferation was assessed 72 h post‐transfection. (A) *NFATC2* mRNA levels in U251 cells after *NFATC2* overexpression. **p < 0.01. (B) Western blotting for *NFATC2* protein in U251 cells after *NFATC2* overexpression. (C) U251 cell images after *NFATC2* overexpression (OE). (D) Cell viability measured by the MTT assay at different culture times and fold change for U251 cells after *NFATC2* overexpression. (E) Observation of U251 cell colonies after 23 days of *NFATC2* overexpression. (F) U251 cells were collected 5 days after *NFATC2* overexpression and the DNA content was determined by flow cytometry with propidium iodide (PI) staining. The percentage of cells in the G1, S and G2/M phases was measured. **p < 0.01. (G) Cells were subjected to flow cytometry with Annexin V‐APC staining. The percentage of apoptotic cells was measured. **p < 0.01.

To verify NFATC2 as the mediator for MRPS16's regulation over glioma cell proliferation, we conducted OE experiments with regard to NFATC2 accompanied by MRPS16 knockdown. We first tested the mRNA and protein level of NFATC2 in U251 cells after plasmid transfected with NFATC2. As can be seen in Figure 5A,B, mRNAs and proteine of NFAT C2 were both highly expressed; notably so for the NFATC2 mRNA where levels were an astounding 7000 fold higher in NFATC2‐OE cells compared to controls. It showed that NFATC2 was indeed overexpressed in the U251 cell line. Figure 5C shows that by fluorescence microscopy, it could be seen that the proliferation of the NFATC2–OE U251 cells was clearly faster than in the controls. NFATC2 overexpression in U251 cells was checked via MTT assay under specified experimental settings, with respect to cell viability. As shown in Figure 5D, the viable cell number on day 3 post transfection with NFATC2. But, the cell viability had significant changes after knocking down MRPS16 in the NFATC2‐OE U251 cells compared with the NFATC2‐OEcells. In the cell colony formation experiment I noticed the same thing. After 23 days of NFATC2 overexpression, the NFATC2‐OE group displayed an obvious increase in the number of U251 cell colonies, as shown in Figure 5E. Nevertheless, when MRPS16 was knocked out in NFATC2's overexpression U251 cell, we noticed the number of colonies sharply decreased. This implies that NFATC2 has an imperative role in promoting proliferation in U251 cells.

In addition, we made a detailed analysis of the cells cycle of U251 with ectopic expression of NFATC2. As shown in Figure 5F, the percentage of NFATC2‐OE U251 cells in the G1 phase was distinctly more than that of control cells, and the percentage of cells in the G2/M phase was significantly reduced compared with the control group, *p* < 0.05, Statistical significance was assessed using Student's *t*‐test. Regarding the S‐phase distribution pattern of NFATC2‐OE U87 cells, it showed great resemblance to that of the control cell population, which indicated that cell population progression seemed to be at the same level. Then after silencing MRPS16 in NFATC2 over—ex pressin U251 cells it was shown there was change to cell cycles distribution this indicates possiable mechanisms for regulation. Compared with NFATC2‐OE cells, NFATC2‐OE + MRPS16‐KD U251 cells showed decreased percentage of G1 phase cells and significantly increased percentage of the G2/M phase cells. So it appears that NFATC2 controls the cell cycle distribution of glioma U251 cells by helping more cells move into the mitosis phase, causing a big increase in how fast glioma cells can grow because of this.

We use flow cytometry for Annexin V—APC staining to check if NFATC2 is involved in the apoptotic pathway of cells. We can see that in Figure 5G. In 5 days after transfection of U251 to NFATC2, when using the dye Annexin V‐APC, it can be clearly seen that the cell population is less, indicating that the apoptosis rate of U251 cells with overexpressed NFATC2 (~4%) compared to the cells transfected with empty plasmids (~6%) was significantly lesser. When *MRPS16* was knocked down in *NFATC2*‐OE U251 cells, the apoptotic cell population increased. These results suggest that *NFATC2* can inhibit glioma cell apoptosis, which may contribute to the positive effect on cell proliferation.

### 3.4 The Wnt/β‐Catenin/ *NFATC2* Cascade May Be Involved in *MRPS16* ‐Induced Glioma Cell Proliferation

Through predictive analysis of data from the KEGG database, we found that *MRPS16* can be associated with *NFATC2* through a variety of signalling pathways. When several signalling pathways with obvious correlation were selected for verification, the Wnt pathway came to our notice. The Wnt pathway and *NFATC2* are interrelated and have obvious regulatory relationships.

**FIGURE 6:**
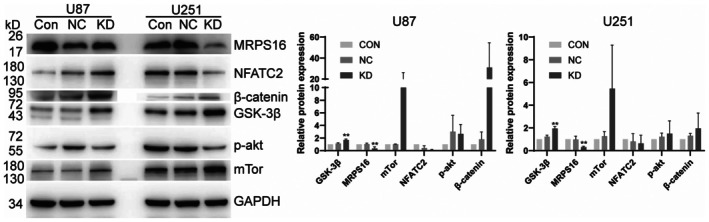
Protein levels for genes in the Wnt/β‐Catenin/*NFATC2* pathway. *MRPS16* was silenced in U87 or U251 cells with shRNA. *MRPS16*, *NFATC2*, β‐Catenin, GSK‐3β, phosphorylated AKT and mTor protein levels were detected by Western blotting.

To investigate whether MRPS16 regulates NFATC2 via the canonical Wnt pathway, MRPS16 was silenced in both U87 and U251 glioma cells, and key components of the Wnt/β‐catenin pathway were subsequently examined. As shown in Figure 6, MRPS16 protein levels were markedly reduced in both cell lines following shRNA‐mediated knockdown. β‐catenin expression was consistently upregulated in both U87 and U251 cells, accompanied by increased levels of GSK‐3β. Statistical significance was assessed using Student's *t*‐test. Notably, NFATC2 expression exhibited divergent trends between the two cell lines, showing an increase in U87 cells but a decrease in U251 cells. These differences likely reflect cell line–specific molecular contexts, as U87 and U251 cells differ in genetic background and baseline signalling activities. Despite these variations, the overall alterations in β‐catenin and GSK‐3β suggest that MRPS16 modulates the activity of the Wnt/β‐catenin/NFATC2 axis.

Also analysed were the proteins within the AKT–mTOR pathway which had previously been linked to MRPS16‐driven gliomagenesis [10]. Similar to our findings in the Wnt pathway, phosphorylated AKT levels were significantly reduced following MRPS16 knockdown in both U87 and U251 cells, while mTOR levels increased in MRPS16‐KD cells (Figure 6).

### 3.5 *MRPS16* Knockdown and *NFATC2* Overexpression Promote Glioma Growth in BALB/c Mice

**FIGURE 7:**
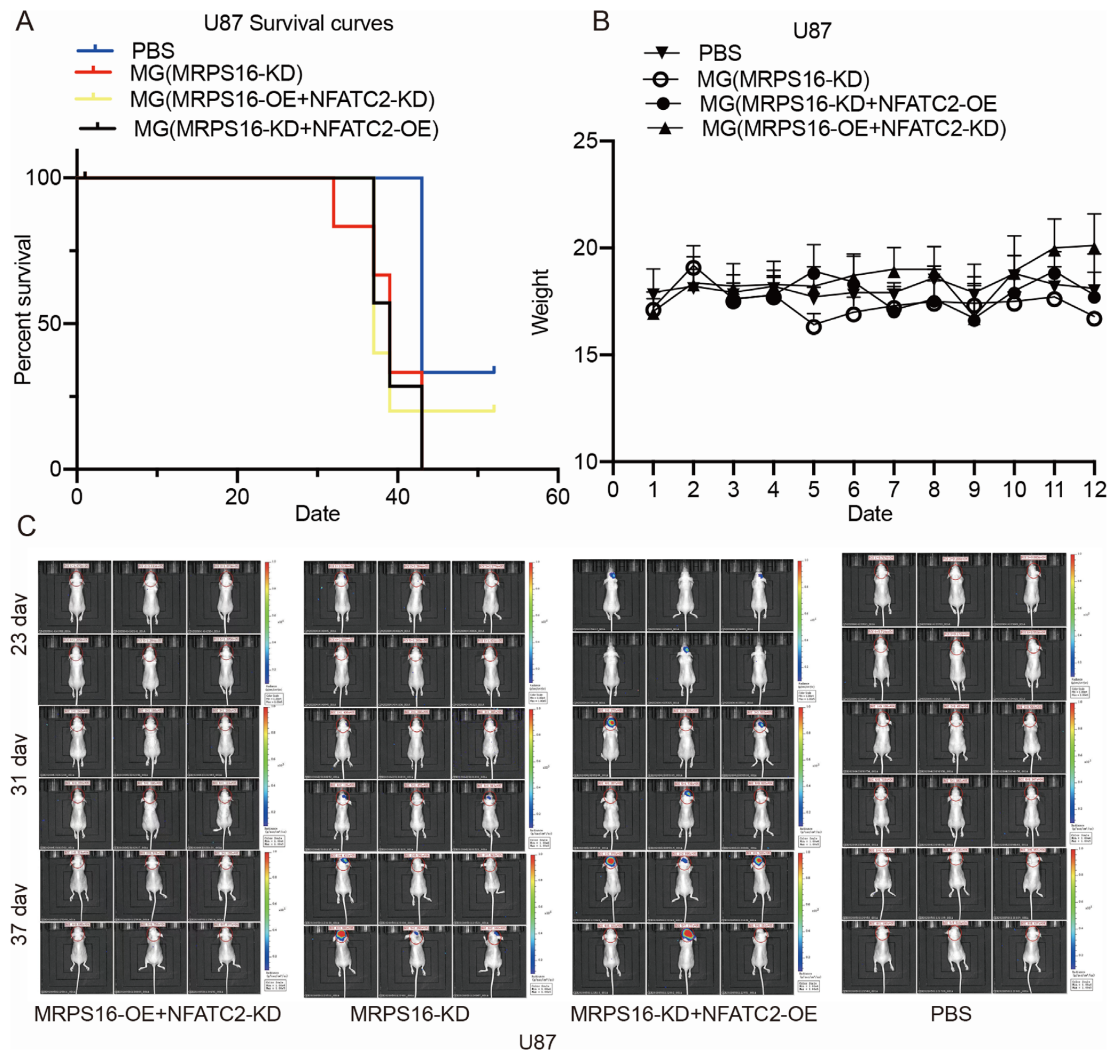
Detection of tumours in BALB/c mice inoculated with U87 cells. BALB/c mice were randomly divided into four groups and inoculated with U87 cells modified with: *MRPS16* knockdown and *NFATC2* knockdown; *MRPS16* knockdown; *MRPS16* knockdown and *NFATC2* overexpression; PBS control. (A) The survival curves of mice inoculated with U87 cells. (B) Body weight of mice inoculated with U87 cells. (C) In vivo images of BALB/c mice taken at 23, 31, 37 days after inoculation.

**FIGURE 8:**
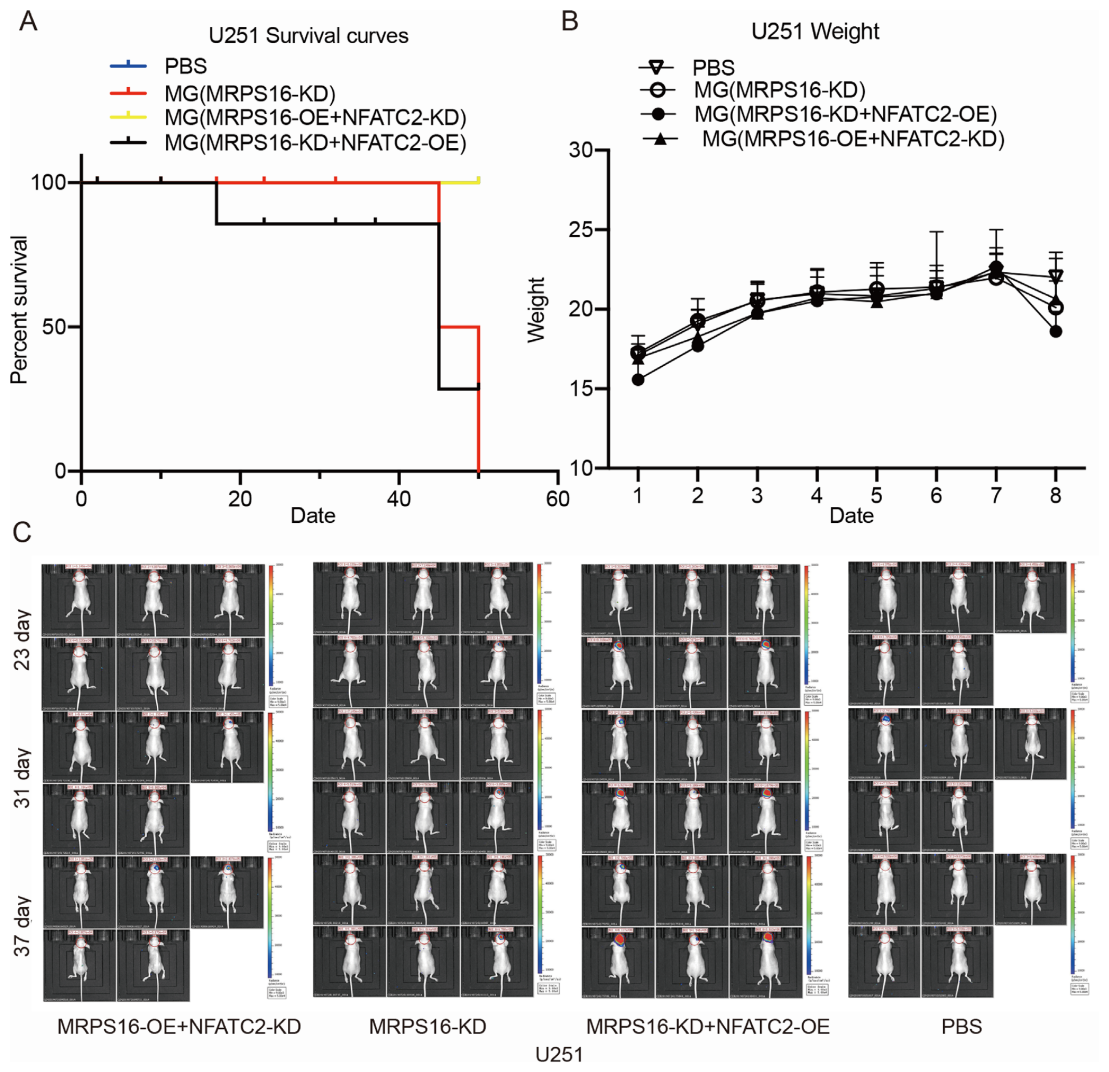
Detection of tumours in BALB/c mice inoculated with U251 cells. Conditions were the same as Figure 6, BALB/c mice were randomly divided into four groups and inoculated with U251 cells modified with: *MRPS16* overexpression and *NFATC2* knockdown; *MRPS16* knockdown; *MRPS16* knockdown and *NFATC2* overexpression; PBS control. (A) The survival curves of mice inoculated with U251 cells. (B) Body weight of mice inoculated with U251 cells. (C) In vivo images of BALB/c mice were taken at 23, 31, 37 days after inoculation.

To further clarify the regulation exerted by MRPS16 and NFATC2 on the proliferation of glioma cells in vivo, U87 cells were injected into the brain of BABL/c mice, and the time of maintenance was more than about 7 weeks. U87 cells were modified with *MRPS16* knockdown, *MRPS16* knockdown and *NFATC2* overexpression or *MRPS16* knockdown and *NFATC2* knockdown. Mice that were inoculated with PBS solution were used as controls. The survival curves are shown in Figure 7A and indicated that all three groups exhibited poor livability compared to the control group. Mice from all groups showed no difference in body weight (Figure 7B). Compared to the *MRPS16*‐KD U87 cells‐inoculated mice, the mice inoculated with *MRPS16*‐KD + *NFATC2*‐OE U87 cells had significantly larger and faster growing tumours, but they did not have statistically affected overall survival. The tumours in the mice inoculated with *MRPS16*‐OE + *NFATC2*‐KD U87 cells grew slower than the other two groups, as indicated by the bioluminescence of the tumour (Figure 7C), and the overall survival is higher than these two groups. In the mice inoculated with U251 cells, the same results were observed (Figure 8). Overall, *MRPS16* and *NFATC2* were shown to promote glioma cell proliferation in BABL/c mice. Statistical significance was assessed using Student's *t*‐test.

## 4 Discussion

As one of the life‐threatening tumours in the brain, malignant gliomas often show dismal prognosis in patients [24, 25]. Along with the advancement in understanding gliomas over recent years, multi‐approach therapies have become more and more important in their treatment, especially the application of targeted therapy for specific cancer‐causing mutation genes [26, 27]. Glioma cell proliferation is critical for tumour malignancy. The identification of a novel molecular target could offer a new approach for glioma treatment. From our study we found out that MRPS16 showed strong upregulation in the samples of glioma tissue. Inhibition of glioma growth and proliferation was largely achieved via promotion of cell cycle stoppage, inducing apoptosis by means of silencing the MRPS16 expression level. On the contrary, it is also observed that overexpression of MRPS16 promotes tumour cell proliferation. Using a tumorigenic model of BABL/c mice. Mice with MRPS16 knockdown exhibited prolonged survival, smaller tumour volumes and less tumour weight compared to those with normal U251 cells implanted into them. So, MRPS16 has a considerable positive correlation with the development of glioma cells, which means it may be worth looking at closely for targeted treatments. Furthermore, it is also possible that the effect of Wnt/β—Catenin/NFATC2 signalling pathway on glioma cell proliferation may occur through MRPS16.

*MRPS16* is a core protein in the mitochondrial respiratory chain, which plays an important role in cell energy supply, cell homeostasis maintenance and related regulation effects [9]. Studies have shown that the assembly process of ribosomes is dependent on the expression of *MRPS16* [8]. Here we validate that MRPS16 does exert regulatory influence over glioma cells, and it is an extensive promotion of proliferation in glioma cells. In glioma cells, we see an increase in the amount of cell death upon the reduction in expression of MRPS16. However, the exact molecular mechanisms and the regulatory cascade are largely unknown.

By detecting and analysing the related downstream genes, *NFATC2* showed high correlation with *MRPS16*. *NFATC2* has a variety of regulatory functions [16], but its role in glioma cells has not been reported before. We knocked down *MRPS16* and found that the expression of *NFATC2* was decreased, suggesting that the expression of *MRPS16* has a positive regulatory effect on *NFATC2*. In in vivo experiments with BABL/c mice inoculated with U251 and U87 cells intracranially, the knockdown of *MRPS16* and overexpression of *NFATC2* led to the tumours in the mice exhibiting a high growth rate compared with mice inoculated with cells that only had the *MRPS16* gene knocked down. When *MRPS16* was overexpressed and *NFATC2* was knocked down at the same time, the previously aforementioned were reversed. By comparison, we have made it clear that *MRPS16* regulates the proliferation of glioma cells through the increased expression of *NFATC2*. However, how *MRPS16* regulates the *NFATC2* expression needs further investigation.

With an even deeper examination of the KEGG database, our attention was drawn to the Wnt signalling pathway as being of particular import. Wnt pathway, which is basically related to both MRPS16 and NFATC2, is an important signally pathway in the regulation of tumour proliferation and progression [28]. We used Western blotting to look at related proteins in the Wnt path when shutting down the expression of MRPS16. In addition to observing GSK—3β increases, we also noticed β—Catenin increases. This is because β—Catenin is an important regulator protein in the Wnt pathway. The complex in the canonical Wnt pathway formed by β‐catenin includes tumour suppressors axin and APC, as well as two types of Ser‐Thr kinases: CK1α/δ and GSK3α/β. CK1α/δ and GSK3α/β play an important role in regulating cellular signalling. In the Wnt signalling pathway, Beta—Catenin acts like the main regulatory element, and its stability is carefully controlled by the destruction complex (DC). Axin is regulated for its stability by the Ubiquitin‐Proteasome System [29]: With the current data and pathway data that was there, I can see a spot for MRPS16 too. An enhancement in the expression of MSIP16 will permit it to bind with the DC part of beta‐catenin thus becoming unstable Phosphorylation of MRPS16 at Ser45 by CK1 causes subsequent phosphorylation at residues Thr 41 and Ser37, and Ser33 by GSK3. As a result, the phosphorylated motif gets converted into a docking site for an F‐box–containing E3 ubiquitin ligase β‐TrCP. And then it will go into the ubiquitin of the axin gene and this causes beta catenin to degrade, lowering the level of beta catenin, when MRPS16 goes down, the receptors bind to it, and that makes the DC move towards the cell membrane, which makes DCs less effective at doing their job. p‐LRP receptor can also directly inhibit GSK‐3, and promote the stability of β‐Catenin in complex by inhibiting the phosphorylation of β‐catenin. The ubiquitination of Phosphorylated β‐catenin will be inhibited in the intact complex. As the amount of Ubiquitin‐Phosphorylated β—catenin increases, the complex is saturated. The freshly made β—Catenin [30] shall now gather, letting it move into the cell nucleus at will. And now that new β—Catenin will bind to the target gene NFATC2 and then inhibit the target gene [31]. Apoptos is would increase while glioma cell proliferation would inhibit. This hypothesis warrants further investigation to clarify the precise molecular mechanisms involved.

It is noteworthy that components of the Wnt/β‐catenin/NFATC2 signalling pathway displayed cell line–specific expression patterns following MRPS16 knockdown. Such differences are common among glioma cell lines due to distinct genetic backgrounds and signalling dependencies. Importantly, these variations do not affect the overall functional conclusions of this study, as both in vitro and in vivo assays consistently demonstrated a pro‐proliferative role of MRPS16.

In the report on *MRPS16* and glioma cell progression, *MRPS16* was shown to facilitate glioma cell progression via the PI3K/AKT/Snail signalling axis [10]. In our study, we also examined this pathway and obtained similar results. Following the knockdown of MRPS16 in U87 and U251 cells, a notable reduction in the phosphorylated AKT level was observed. Furthermore, we found that the level of mTOR increased in MRPS16‐KD cells. The role of these proteins in glioma cell proliferation will be further elucidated through additional investigation in the future. In the clinical treatment of patients, the potential side effects associated with MRPS16 blockade should also be carefully considered.

In conclusion, our results showed that MRPS16 is a novel oncogene related to glioma proliferation. High expression of MRPS16 was displayed in human glioma, and it was connected with the poor prognosis of glioma. MRPS16 can promote glioma tumour proliferation and inhibit glioma cell apoptosis, so it might be supposed that MRPS16 could control the multiplication of glioma cells by way of the way in which Wnt/β—Catenin/NFATC2. In order to inhibit GDN4 and thus target glioma, the targeting of MRPS16 might be a new approach to treat glioma.

## Author Contributions

X.L., X.W. and X.J. designed the research; X.L. and S.Y. conducted experiments; X.L, S.Y., M.W., Z.G. and Q.C. analyzed the data; X.L. and S.Y. wrote and revised this manuscript; X.W. and X.J. revised the manuscript and supervised the research. The authors read and approved the final manuscript.

## Funding

The authors have nothing to report.

## Ethics Statement

The animal experiments were approved by the Animal Management and Use Committee of Union Hospital, Tongji Medical College, Huazhong University of Science and Technology, Hubei, China.

## Consent

Written informed consent was obtained from all patients or their legal guardians prior to tissue collection. The study protocol was reviewed and approved by the Ethics Committee of Union Hospital, Tongji Medical College, Huazhong University of Science and Technology.

## Conflicts of Interest

The authors declare no conflicts of interest.

## Data Availability

The data that support the findings of this study are available from the corresponding author upon reasonable request.
